# The Role of Neck Musculature in Traumatic Brain Injuries in Older Adults: Implications From Sports Medicine

**DOI:** 10.3389/fmed.2019.00053

**Published:** 2019-03-27

**Authors:** Tyler A. Wood, Steven Morrison, Jacob J. Sosnoff

**Affiliations:** ^1^Department of Kinesiology and Community Health, University of Illinois at Urbana-Champaign, Urbana, IL, United States; ^2^School of Physical Therapy and Athletic Training, Old Dominion University, Norfolk, VA, United States

**Keywords:** accidental falls, head movements, neck muscles, older adults, traumatic brain injury

## Abstract

Traumatic brain injuries (TBIs) are common and serious injuries to older adults. The majority of TBIs in older adults are sustained when the head impacts the ground or other surface during a fall. While several non-modifiable risk factors have been identified for fall-related TBIs in older adults, there still remains a dearth of knowledge surrounding modifiable risk factors. Thus, this significant knowledge gap warrants an investigation into research across disciplines. The sports medicine literature has examined several modifiable risk factors to prevent a mild form of TBI known as concussion. While this research has identified several risk factors, one particular risk factor may have potential implications to fall-related TBIs in older adults. The sports medicine literature has shown that decreased neck strength and slower neck muscle activation are significant predictors for sports-related concussion. Similarly, older adults experience age-related declines to neck muscle strength and muscle activation. Consequently, these age-related declines to the neck musculature may result in the inability of older adults to control their head during a fall, which results in greater impact forces being transmitted to the brain and increases the risk of TBI. This perspective article assesses the sports medicine literature related to the implications of neck strength and muscle activation in sports-related concussion, discusses age-related declines to neck strength and muscle activation, and highlights the potential impact of the neck musculature on fall-related TBIs in older adults.

## Introduction

Traumatic brain injuries (TBIs) are induced by biomechanical forces that are transmitted to the brain after a direct blow to the head, face, neck, or body (1). In 2013, the Centers for Disease Control and Prevention reported that there were approximately 2.8 million TBIs that resulted in emergency department visits, hospitalizations, and/or deaths in the United States (1). Furthermore, the rate of TBIs in older adults almost doubled between 2007 and 2013 (1). For older adults, the health consequences of a TBI are more marked with extended hospitalizations and a >10% fatality rate (2). Upwards of 80% of TBIs in older adults are a result of the head hitting the ground or other surface during a fall (2).

There is little doubt that fall-related TBIs are a major cause of morbidity and mortality in older adults (1–3). Several investigations have identified non-modifiable risk factors, such as gender and age, as major mitigating factors in fall-related head injuries (1–7) However, despite our understanding of the significance of this problem, there has been limited research examining modifiable risk factors of fall-related TBIs in older adults (1–7). One report concluded that older adults should attempt to avoid fall-related head impact but provided minimal insight on how to accomplish this feat (4).

As there is minimal information on modifiable risk factors for fall-related TBIs in older adults, it is worthwhile to examine research across disciplines for insight. The sports medicine literature has focused on the prevention of a mild form of TBI, known as concussion (8). Indeed, it has been estimated that young adults experience approximately 1.6–3.8 million sports-related concussions each year in the United States (9). The sports medicine literature has examined several potentially modifiable risk factors including the use of protective equipment, rules and legislation governing sport, and implications of the neck musculature (8, 10, 11). While the majority of modifiable risk factors may not pertain to older adults, the implications of the neck musculature as it relates to sports-related concussion may provide valuable information.

Prior to discussing the implications of neck musculature for mild TBI, it is important to understand the location and action of the primary movers of the neck musculature. The neck musculature consists of sternocleidomastoids (SCM), splenius capitis, and upper trapezius muscle (12). The SCM originates on the manubrium and the medial end of the clavicle; it then inserts on the mastoid process (12, 13). Unilateral activation of the SCM results in lateral flexion while bilateral activation results in neck flexion occurs (12, 13). The splenius capitis originates on the lower half of the ligamentum nuchae and spinous processes of C7-T3. Unilateral activation of the splenius capitis results in lateral flexion while bilateral activation results in neck extension. The trapezius is the most superficial muscle in the back. It originates from the superior nuchal line, external occipital protuberance, ligamentum nuchae, and the spinous processes of C7 to T12 (12, 13). The upper trapezius extends the neck, along with contributing to lateral flexion and rotation (12, 13). There are many other deep muscles located on the posterior and lateral sides of the neck and attach to individual vertebrae (12). These smaller muscles are beyond the scope of this review.

In general, the activation of the neck musculature has been shown to decrease head acceleration and potentially prevent sports-related concussions (11, 14–30). Specifically, it has been demonstrated that decreased neck strength and slower activation of the neck muscles are a significant predictor for sports-related concussion. Empirical data has also revealed that stronger neck muscles and faster muscle activation mitigates forces at head impact (11, 16, 31).

Although it would be logical to speculate that neck strength and muscle activation are related to the magnitude of head acceleration experienced during a fall and TBI in older adults, there is a dearth of evidence. Thus, the purpose of this perspective article is to examine the sports medicine literature surrounding the implication of neck strength and muscle activation in sports-related concussion, discuss age-related changes to neck strength and muscle activation, and highlight the potential impact on fall-related TBIs in older adults.

## Literature Search

A keyword search was performed in PubMed, Cumulative Index to Nursing and Allied Health Literature (CINAHL), Web of Science, and Ovid-Medline. The search algorithm included all possible combinations of the keywords from the following three groups: (1) “concussion,” “traumatic brain injury,” and “head injury;” (2) “neck” and “cervical;” and (3) “muscle strength,” “muscle activation,” and “impact velocity.” A reference list search (i.e., backward reference search) and cited reference search (i.e., forward reference search) were also conducted based on the full-text articles that met the study selection criteria that were identified from the keyword search. Articles identified from the backward and forward reference search were further screened and evaluated using the same study selection criteria. Reference searches were repeated on all newly identified articles until no additional relevant articles were found. Articles published up to March 10, 2018 were identified.

Studies that met all the following criteria were included in the review: study design: randomized controlled trial, case control study, cohort study, pre-post-study, or cross-sectional study; study subjects: human male and female participants of all ages; main outcome: neck strength and neck activation related to head movement or control and TBIs; article type: peer-reviewed publication; and language: English.

**Figure 1:**
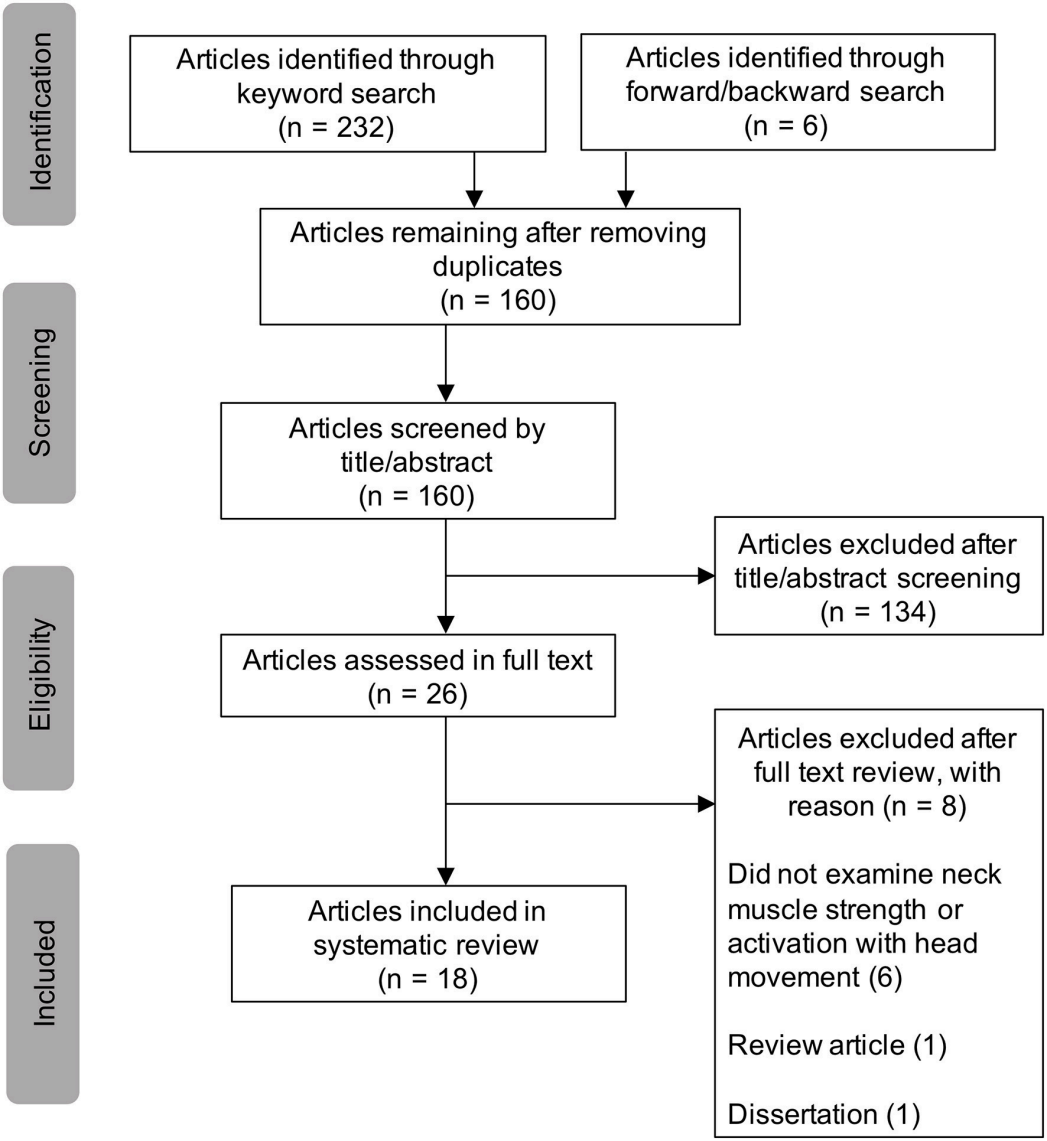
Study selection flowchart.

T. Wood conducted the literature search. Figure 1 displays the study selection flowchart. A total of 160 unduplicated articles were identified through the keyword and reference search, by which 134 articles were excluded by title and abstract screening. Twenty-six articles were assessed in full texts, in which 18 articles were identified and included in the review (11, 14–30). Eight articles were excluded after full text review. Six studies were excluded because they did not examine neck strength or activation with head movement (32–37). One study was excluded because it was a review (38) and another was excluded because it was a dissertation (39). Table 1 reports the basic characteristics of each of the included studies.

**Table 1:** Basic characteristics of the included studies.

**Author**	**Study design**	**Sample size and gender**	**Sample age [mean (SD)]**	**Neck strength or muscle activation**	**Protocol summary**	**Summary of findings**
Bauer et al. (14)	Cross-sectional	15 0 males; 15 females	20.3 (2.3)	Activation	Recorded EMG activity of the SCM and trapezius muscles during soccer headings.	SCM and trapezius play a major role to stabilize the head; greater muscle activity resulted in greater stability of the head/neck complex.
Bretzin et al. (15)	Cross-sectional	13 5 males; 8 females	19.8 (0.9)	Strength	Correlated head-neck anthropometrics and neck strength to head linear acceleration and rotational velocity during soccer headings. Neck strength was measured with a hand-held dynamometer in FL, EX, LLF, RLF, RO.	Neck girth negatively correlated to linear acceleration and rotational velocity. Stronger neck muscles muscle resulted in lower linear acceleration.
Choi et al. (16)	Cross-sectional	8 3 males; 5 females	26.5 (5.3)	Activation	Measured SCM activity during backwards falls.	SCM activity was greater during hard head impact scenarios, which indicated SCM activation could mitigate head impact severity.
Collins et al. (11)	Prospective cohort	6,704 3,588 males; 2,895 females	NR	Strength	Used a validated hand-held tension scale to assess neck strength in FL, EX, LLF, RLF. Concussion incidence and athlete exposure data was recorded throughout the season.	Overall neck strength was a significant predictor of concussion after adjusting for gender and sport.
Dezman et al. (17)	Cross-sectional	16 8 males; 8 females	20.5 (1.9)	Strength	Measured neck strength in FL and EX with a spring-type clinical dynamometer. Correlated neck strength imbalance with angular and linear head acceleration during soccer heading	Greater neck strength imbalance correlated to greater angular head acceleration, but not linear acceleration.
Eckner et al. (18)	Cross-sectional	46 24 males; 22 females	16.3 (5.0)	Strength and activation	Measured neck strength with an inline force transducer in FL, EX, LLF, and right axial RO. Correlated neck strength with peak linear and angular head velocity from baseline and anticipatory cervical muscle activation conditions during perturbation testing.	Greater neck strength and anticipatory activation was associated with decreased peak linear and angular velocities.
Eckner et al. (19)	Pre-post	17 15 males; 2 females	14.6 (1.9)	Strength	Measured neck strength with an inline force transducer in FL, EX, LLF, and right axial RO. Participants completed either a manual resistance neck strengthen program with a general resistance training program (*n* = 13) or only a resistance training program (*n* = 4) as a control. Linear and angular head velocity was observed with perturbation.	The neck strengthening group saw a greater increase in neck strength. Both groups saw decreases in head linear and angular velocity after training during perturbation. The neck strengthening group had greater decreases in linear head velocity and the control group had greater decreases in angular head velocity
Gilchrist et al. (20)	Cross-sectional	25 25 males; 0 females	25.6 (NR)	Strength	Measured neck strength with a Multi-Cervical Unit in FL, EX, LLF, RLF, 45° of left and right FL, and 45° of left and right extension with the head in neutral and flexed at 20 degrees.	When the neck was flexed to 20 degrees, it was found that there was a lower moment generating capacity of the neck in the first 50 ms of force application. This finding indicates that with head flexion, less force can be produced to resist head impact.
Gutierrez et al. (21)	Cross-sectional	17 0 males; 17 females	15.9 (0.9)	Strength	Measured neck strength with a handheld dynamometer in FL, EX, LLF, and RLF. Neck strength was correlated with head acceleration during soccer heading.	There was a moderate negative correlation between all directions of neck strength and head acceleration.
Ito et al. (22)	Cross-sectional	13 11 males; 2 females	range = 21 to 49	Activation	Examined EMG activity of the SCM in healthy subjects and labyrinthine-defective subject during head free fall	Labyrinthine-defective had delayed SCM EMG activity
Ito et al. (23)	Cross-sectional	16 13 males; 3 females	range = 23 to 68	Activation	Examined EMG activity of the SCM in healthy subjects and labyrinthine-defective subject during head free fall under two conditions: fall passively or actively right the head as quickly as possible.	Under the passive condition, normal subjects had an SCM latency of 24.5 ms compared to 67.4 ms in the labyrinthine-defective subjects. Under the active righting position, there was no difference in EMG activity between the groups.
Kuramochi et al. (24)	Cross-sectional	9 9 males; 0 females	22.6 (4.1)	Activation	Examined SCM EMG activity under an anticipated head blow stimuli and an unanticipated head blow stimuli	Under the unanticipated condition, there was significantly greater SCM EMG activity, yet there were no differences in head acceleration during the conditions.
Lisman et al. (25)	Pre-post	16 16 males; 0 females	21.6 (2.8)	Strength and activation	Neck strength was assessed with a digital force gauge in FL, EX, LLF, and RLF. EMG during tackling was also assessed. Participants then completed an 8 week cervical resistance training program.	Cervical resistance training resulted in an increase in isometric cervical extension and left lateral flexion strength, but had no did not result in differences in SCM and trapezius EMG response, peak linear head acceleration, or angular head acceleration during tackling from before training
Mansell et al. (26)	Pre-post	36 17 males; 19 females	19.2 (0.9)	Strength and activation	Measured neck strength with a handheld dynamometer in FL and EX. EMG was measured in response to a perturbation. Participants were randomized into a control group and an 8 week cervical resistance training group.	Only female participants had strength gains due to the resistance training program; isometric extension strength increased by 22.5%. However, there were no changes in head kinematics, EMG activity, or stiffness after training in males or females.
Mihalik et al. (27)	Prospective cohort	37 Gender NR	15.0 (1.0)	Strength	Neck strength was measured with a handheld dynamometer in FL, EX, LLF, RLF, and RO before youth ice hockey season and head impact acceleration was assessed.	There were significant differences in neck strength, yet there were no differences in linear or angular head acceleration.
Schmidt et al. (28)	Prospective cohort	49 Gender NR	17.8(1.1)	Strength	Neck strength was measured using the HUMAC NORM Testing and Rehabilitation System in FL, EX, LLF, and RLF. Participants then underwent cervical perturbation.	Participants with stronger neck muscles did not have reduced head impacts. Participants with greater cervical stiffness and less angular displacement after perturbation did have reduced odds of higher magnitude head impacts.
Simoneau et al. (29)	Cross-sectional	7 4 males; 3 females	23.5 (NR)	Activation	Participants had varying amounts of head preloading. The head was unexpectedly moved forward or backward with an additional weight.	With greater amounts of preloading, there was greater stiffness and viscosity, which lead to lower peak angular velocity.
Tierney et al. (30)	Cross-sectional	40 20 males; 20 females	25.3 (4.2)	Strength and activation	Neck strength was measured with a handheld dynamometer in FL and EX, along with EMG of the SCM and trapezius muscle to Examined gender differences of head-neck segment dynamic stabilization in response to perturbation.	Females had greater head-neck segment angular acceleration. This finding could be due to significantly less isometric neck strength, neck girth, and head mass, which resulted in lower levels of head-neck segment stiffness.

*EMG, electromyography; EX denotes extension; FL, flexion; LLF, left lateral flexion; NR, not reported; RLF, right lateral flexion; RO, rotation; SCM, sternocleidomastoid*.

## Neck Strength and Muscle Activation Implications in Older Adults

### Neck Strength as Risk Factor for Fall-Related TBI

Neck strength is a significant predictor of concussions in high school athletes. In a large epidemiological study of 6,704 high school athletes, Collins et al. (11) reported that lower overall neck strength was a significant predictor of sports-related concussion. Within this investigation of 6,704 high school athletes, researchers utilized a valid and reliable hand-held tension scale to measure strength in neck flexion, extension, and right/left lateral flexion. An average of the four strength measures was utilized in analysis. This finding was supported by several smaller investigations utilizing various measures of neck strength, which empirically revealed that greater neck strength resulted in significantly less head acceleration in response to perturbations in young athletes (15, 17–21, 30).

It is well established that with normal aging there are declines to muscle strength (40–42). Between the ages of 20 and 60, decreases between 35 and 45% have been reported for neck flexion and extension (43). Although there is limited data, it is likely that the general decline in neck strength continues into advanced age. The consequences of this progressive decline in neck strength may be particularly problematic for older adults at risk of falling. For example, with decreases in neck strength, older individuals may be unable to appropriately support their head during a fall leading to greater head acceleration at head impact and thus, have an increased risk of suffering a TBI.

### Neck Muscle Activation as Risk Factor for Fall-Related TBI

It is important to note that not all empirical investigations of neck strength and head acceleration observed a strong association between these factors. Mihalik et al. (27) found that increased neck strength did not result in lower head acceleration during impact in youth ice hockey; this finding could potentially be due to the age and competitive level of the sample. Conversely, Schmidt et al. (28) found that it was not neck strength, but neck stiffness that contributed to decreased injury risk. These finding highlights that neck strength alone may not fully characterize the neuromuscular control of the head necessary to mitigate head impact during a fall. It has been proposed that stabilization of the head during a perturbation results from the rapid activation of neck musculature to circumvent the perturbation, similar in context to whole body postural responses following external challenges to balance (28, 29, 44).

Activation of the SCM and the upper trapezius muscles are important for head stabilization and reducing head impact severity in young adults (14, 16, 22, 23, 28–30). A previous investigation specifically assessed the activation of the SCM muscles during simulated backwards falls in healthy young adults (16). For this study, participants fell under three different head control instructions. These were: (1) no impact—prevent the head from impacting the ground, (2) minimal impact—allow the head to impact the ground but with minimal force, and (3) hard impact—allow the head to impact the ground and inhibit efforts to reduce impact. During the no impact and minimal impact conditions, the SCM played a significant role in supporting the head. For the hard impact condition, the SCM was minimally active, which indicates that activation of the SCM most likely contributes to the prevention and modulation of head impact during a backward (16).

In young adults, the SCM has similar amounts of Type I and Type IIa muscle fibers (45). With aging, the composition of the SCM remodels to takes on a slower muscle phenotype (44–47). The area of fast twitch decrease and the number of slow twitch fibers increase, which result in slower SCM activation (44–47). Although there is limited data, it is logical to speculate that alterations to motor unit population occur in the splenius capitis and upper trapezius. The consequences of the age-related changes in neck muscle properties may mean that older adults react slower to external destabilizing perturbations. As a result, they are unable to quickly stabilize their head during a fall, which increases the possibility of TBI.

### Neck Strength and Muscle Activation Interventions

Given the link between neck strength and head motion, several studies have examined the effectiveness of exercise programs targeting the muscles around the neck/head complex for improving overall head control. These previous investigations found mixed results. It was found that resistance training in college aged athletes did not alter EMG activity or head kinematics during impact (25, 26). Conversely, in a different adolescence sample, a resistance program successfully altered head kinematics during simulated impact (19). The differences observed in these studies may relate to the age and skill level of the participants. In addition to resistance training, it has also been suggested that neuromuscular training designed to enhance the neck muscles' dynamic responses to perturbation may be more beneficial than resistance training alone (28). While, resistance training has been showed to improve strength in upper and lower extremities in healthy older adults (48, 49), there is very limited empirical data pertaining to the efficacy of neck strength resistance programs in older adults. It remains to be seen if neck strength resistance programs can result in improvements in head control.

## Future Directions

While the sports medicine literature highlights the potential implications of changes in neck strength and muscle activation patterns to mitigate head impact forces, these studies were primarily conducted on young healthy adults in relation to sport concussion. Although this body of research leads to the speculation that decreases in neck strength and muscle activation may be a significant contributor to fall-related TBIs in older adults, there are significant knowledge gaps. These knowledge gaps include age-related changes to neck muscle strength after the age of 75 years, age-related strength changes in all directions of motion, and age-related changes to muscle activation of the SCM, upper trapezius, and splenius capitis in response to a perturbation. It is also not clear if neck muscle strength or function is related to head acceleration in older adults. It should be further noted that obvious differences exist between the mechanics of sports concussion and those related to fall-induced TBIs. Consequently, more research is needed to understand the mechanisms of fall-related TBIs and the specific role of neck strength and muscle activation patterns for stabilization of the head during a fall in older adults. While promising, neck strength and muscle activation may together be described as a single factor in the multifactorial problem of fall-related TBIs. Other potential factors include polypharmacy, the use of antiarrhythmics, and unsafe bed or chair transfers (4). Investigations of the relationship between neck strength, muscle activation, and head acceleration during falls in older adults is warranted.

## Author Contributions

TW was the primary author who researched the background information and wrote the manuscript. SM helped to organize the manuscript and added information on age-related declines to the neck musculature. JS assisted in organizing the manuscript and added input throughout the manuscript.

### Conflict of Interest Statement

The authors declare that the research was conducted in the absence of any commercial or financial relationships that could be construed as a potential conflict of interest.
